# Prosthetic valve endocarditis caused by *Candida lusitaniae,* an uncommon pathogen: a case report

**DOI:** 10.1186/1752-1947-3-7611

**Published:** 2009-05-14

**Authors:** Ross G Michel, Gary T Kinasewitz, Douglas A Drevets, Jeremy H Levin, Douglas W Warden

**Affiliations:** 1 Division of Pulmonary/Critical Care Medicine and Infectious Diseases, University of Oklahoma Health Sciences Center, Oklahoma City, Oklahoma, USA; 2 Division of Infectious Diseases, University of Oklahoma Health Sciences Center, Oklahoma City, Oklahoma, USA; 3 Department of Pathology, University of Oklahoma Health Sciences Center, Oklahoma City, Oklahoma, USA

## Abstract

**Introduction:**

*Candida lusitaniae* was originally described as a human pathogen in 1979 and typically affects immunocompromised patients.

**Case presentation:**

We describe a case of prosthetic valve endocarditis with *Candida lusitaniae* in an immunocompetent 62-year-old woman following aortic valve replacement. *In vitro* testing demonstrated that our isolate was sensitive to amphotericin B, caspofungin and fluconazole.

**Conclusion:**

The infection was lethal despite aggressive medical and surgical management and sterilization of blood cultures. The outcome of our case illustrates the need to recognize *Candida lusitaniae* fungemia as a life-threatening infection in a patient with a prosthetic aortic valve.

## Introduction

*Candida* species are the most frequent causes of fungal endocarditis. Mortality from *Candida* endocarditis has been reported at a rate of 50-80% [1]. *Candida albicans* is the most frequently isolated species, followed by *C. parapsilosis*, *C. glabrata* and *C. tropicalis*. *C. lusitaniae*, originally described as a human pathogen in 1979, typically affects immunocompromised patients and accounts for less than 5% of all invasive *Candida* infections [2,3]. Infections including meningitis, osteomyelitis and peritonitis have been reported, but the most common presentation of this pathogen is fungemia in patients undergoing chemotherapy for cancer [4]-[6]. Prosthetic valve endocarditis with this organism has been described only once previously [7]. Although resistance to amphotericin B has been reported, a recent susceptibility study has shown that the majority of *C*. *lusitaniae* isolates are flucytosine resistant and amphotericin-B susceptible [8]. In contrast, in vitro testing of the isolate in this case demonstrated sensitivity to amphotericin B, caspofungin and fluconazole.

## Case presentation

A 62-year-old Caucasian woman presented with a two-month history of intermittent fever. Her past medical history included hypertension, dyslipidema, coronary artery disease and mild chronic kidney disease. She was found to have *Enterococcus faecalis* endocarditis of her native, bicuspid, aortic valve. She was treated with vancomycin, gentamicin and penicillin and subsequently underwent aortic valve replacement (AVR) with a Saint Jude valve in October of 2004. Her postoperative course was complicated by evacuation of a mediastinal hematoma, splenectomy and a partial colectomy with diverting colostomy for hemorrhagic colitis. The patient had been discharged and re-admitted multiple times and was ultimately transferred to the Oklahoma University Medical Center in May 2005.

Upon transfer, the patient complained of dyspnea, orthopnea and lower-extremity swelling. She was afebrile and hemodynamically stable. Her physical examination revealed jugular venous distention, a III/VI systolic murmur in the right second intercostal space, and bilateral lower-extremity edema to the knees. No peripheral stigmata of endocarditis were identified.

**Figure 1:**
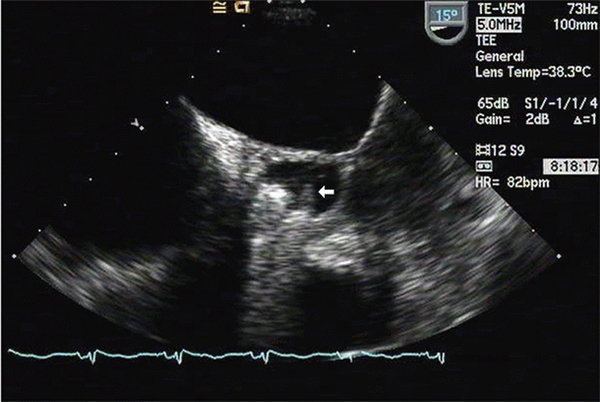
**Trans-esophageal echocardiographic image of prosthetic aortic valve vegetations**. White arrow indicates largest fungal vegetation identified in this case.

**Figure 2:**
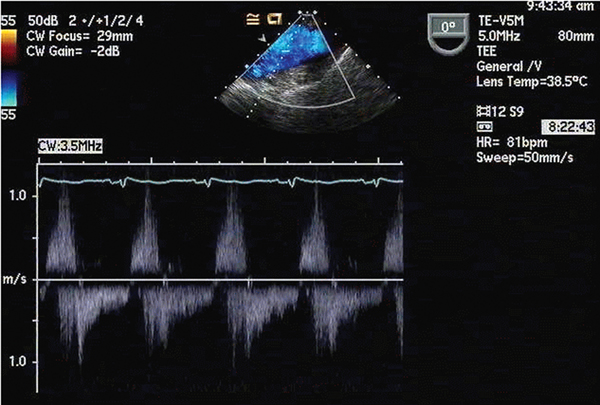
**Infected prosthetic aortic valve regurgitation**. Trans-esophageal echocardiography was helpful in identification of the prosthetic valve infection and dysfunction.

**Figure 3:**
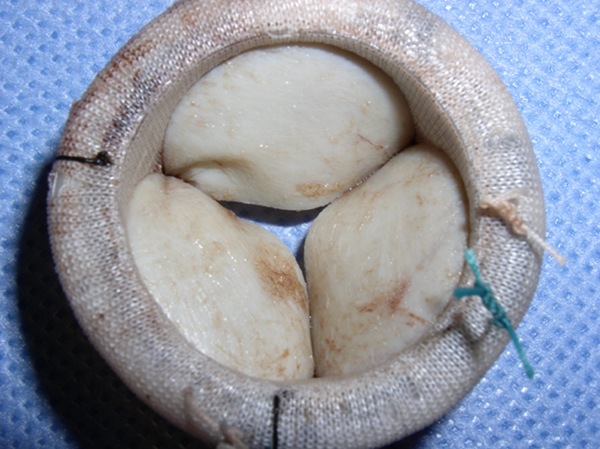
**Infected prosthetic aortic valve**. Notice the irregularities on the leaflet surfaces of the removed prosthetic valve.

**Figure 4:**
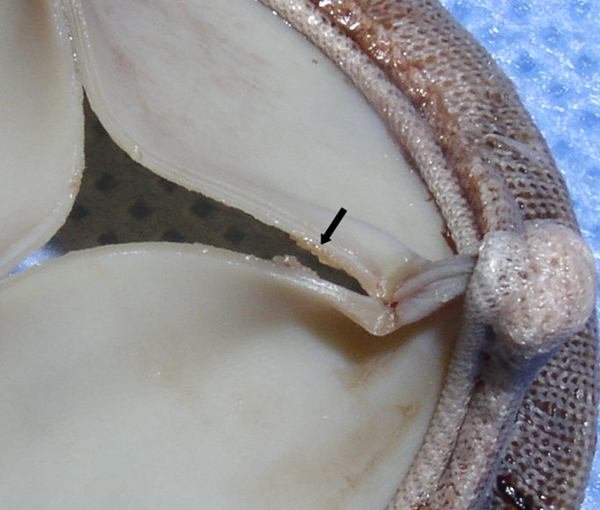
**Prosthetic aortic valve vegetation**. Black arrow indicates a vegetation on the removed prosthetic valve.

Nine blood cultures obtained over a five-day period were positive for *Candida lusitaniae*. Susceptibility testing was performed on the initial isolate only and revealed sensitivity to amphotericin B, caspofungin and fluconazole (Table 1). Transesophageal echocardiography revealed multiple vegetations on a partially dehiscent mechanical aortic valve (Figure 1) with severe aortic valvular regurgitation and a left ventricular ejection fraction of 50% (Figure 2). Therapy with caspofungin was initiated prior to obtaining results of susceptibility testing on the initial isolate and was continued based on the minimal inhibitory concentration (MIC). The patient eventually underwent repeat AVR with coronary artery bypass grafting. Vegetations were noted on her removed prosthetic valve (Figures 3 and 4). Her post-operative course was complicated by a chest hematoma which was evacuated. Despite eventual clearance of her fungemia, the patient died from multi-organ failure in June 2005.

**Table 1:** MICs and minimum fungicidal concentrations for *Candida lusitaniae* isolate from May 2005

Agent	Parameter, value (mcg/mL)
Amphotericin B	MIC 0.50
	MFC 0.50
Caspofungin	MIC 0.06
	MFC ND
Fluconazole	MIC 0.25
	MFC ND

**Note:** Susceptibility of blood culture isolate to Caspofungin performed by the Fungus Testing Lab, UTHSC, San Antonio, TX. All other susceptibilities of blood culture isolate were determined by Microbiology Laboratory at OU Medical Center, Oklahoma City, OK.

**MFC** = minimum fungicidal concentration. **ND** = Not done.

## Discussion

It remains unknown whether the source of our patient's fungemia was her abdominal surgery or a central venous catheter infection. Transesophageal echocardiography was helpful in identifying prosthetic valve infection and dysfunction. MIC testing of the initial fungal isolate demonstrated susceptibility to multiple antifungals. Potential biofilm formation on the prosthetic valve could have resulted in a discrepancy between the MIC derived from the blood isolate and the isolate at the site of infection, although the latter was not submitted for testing. Aggressive surgical management and antifungal treatment led to clearance of our patient's fungemia. Nevertheless, the infection resulted in a lethal outcome as in the previously described case [7]. The shared outcome of our case with that of the previous case clearly illustrates the need to recognize *Candida lusitaniae* fungemia as a life-threatening infection in a patient with a prosthetic aortic valve.

## Conclusion

We describe a case of prosthetic valve endocarditis with *Candida lusitaniae* following AVR in an immunocompetent patient. To our knowledge, this is only the second reported case of prosthetic valve endocarditis due to this uncommon *Candida* species. *In vitro* testing demonstrated that our isolate was sensitive to amphotericin B, caspofungin and fluconazole. As in the previously described case, this infection was lethal despite aggressive medical and surgical management and sterilization of blood cultures.

## Consent

Written informed consent was obtained from the patient's next-of-kin for publication of this case report and accompanying images. A copy of the written consent is available for review by the Editor-in-Chief of this journal.

## Competing interests

The authors declare that they have no competing interests.

## Author's contributions

All authors have made substantial contributions to the case presentation and discussion, have been involved in drafting the manuscript or revising it critically for important intellectual content and have given final approval of the version to be published.
